# Hemodynamic Effects of Anthrax Toxins in the Rabbit Model and the Cardiac Pathology Induced by Lethal Toxin

**DOI:** 10.3390/toxins3060721

**Published:** 2011-06-23

**Authors:** William S. Lawrence, Jeffrey R. Marshall, Diana L. Zavala, Lori E. Weaver, Wallace B. Baze, Scott T. Moen, Elbert B. Whorton, Randy L. Gourley, Johnny W. Peterson

**Affiliations:** 1 Department of Microbiology and Immunology, University of Texas Medical Branch, Galveston/TX 77555, USA; Email: jrmarsha@utmb.edu (J.R.M.); stmoen@utmb.edu (S.T.M.); jpeterso@utmb.edu (J.W.P.); 2 Animal Resources Center, University of Texas Medical Branch, Galveston/TX 77555, USA; Email: dlzavala@utmb.edu (D.L.Z.); leweaver@utmb.edu (L.E.W.); drrandy_706@yahoo.com (R.L.G.); 3 Department of Veterinary Sciences, University of Texas MD Anderson Cancer Center, Bastrop/TX 78602, USA; Email: wbaze@mdanderson.org; 4 Institute of Human Infections, University of Texas Medical Branch, Galveston/TX 77555, USA; Email: ewhorton@utmb.edu

**Keywords:** anthrax, lethal toxin, rabbit model, cardiac

## Abstract

Anthrax lethal toxin (LeTx) and edema toxin (EdTx) have been shown to alter hemodynamics in the rodent model, while LeTx primarily is reported to induce extensive tissue pathology. However, the rodent model has limitations when used for comparison to higher organisms such as humans. The rabbit model, on the other hand, has gained recognition as a useful model for studying anthrax infection and its pathophysiological effects. In this study, we assessed the hemodynamic effects of lethal toxin (LeTx) and edema toxin (EdTx) in the rabbit model using physiologically relevant amounts of the toxins. Moreover, we further examine the pathological effects of LeTx on cardiac tissue. We intravenously injected Dutch-belted rabbits with either low-dose and high-dose recombinant LeTx or a single dose of EdTx. The animals’ heart rate and mean arterial pressure were continuously monitored via telemetry until either 48 or 72 h post-challenge. Additional animals challenged with LeTx were used for cardiac troponin I (cTnI) quantitation, cardiac histopathology, and echocardiography. LeTx depressed heart rate at the lower dose and mean arterial pressure (MAP) at the higher dose. EdTx, on the other hand, temporarily intensified heart rate while lowering MAP. Both doses of LeTx caused cardiac pathology with the higher dose having a more profound effect. Lastly, left-ventricular dilation due to LeTx was not apparent at the given time-points. Our study demonstrates the hemodynamic effects of anthrax toxins, as well as the pathological effects of LeTx on the heart in the rabbit model, and it provides further evidence for the toxins’ direct impact on the heart.

## 1. Introduction

*Bacillus anthracis*, the causative agent of anthrax, brings about extensive pathophysiology during the course of human infection, and it is associated with an extremely high mortality rate if either no treatment is administered or treatment is delayed [1,2,3,4]. The bacteria secrete three proteins known as protective antigen (PA), lethal factor (LF), and edema factor (EF). These proteins combine to form two exotoxins, lethal toxin (LeTx; PA and LF) and edema toxin (EdTx; PA and EF) [5]. LF is a zinc-metalloprotease that cleaves all but one mitogen activated protein kinase kinase (MAPKK) [6,7,8], while EF is a high rate calmodulin-dependent adenylate cyclase that produces an abundance of intracellular cAMP [6,8,9]. Upon binding to target receptors on host cells, monomers of PA, the receptor-binding moiety, heptamerize and bind three units of LF and/or EF. The entire complex then undergoes endocytosis, forming an endosome that later acidifies and releases the units of LF and EF into the cytosol [8,10,11]. Here, LF and EF is capable of altering cellular physiology and/or inducing cell death [11,12,13].

Past reports have highlighted the contributions of LeTx and EdTx to the overall pathophysiology associated with anthrax infection [14,15,16,17,18,19,20], with some elucidating the cardiovascular effects [21,22,23]. The toxins have been shown to bring about distinct hemodynamic outcomes that are believed to be partially responsible for the sequelae associated with inhalational anthrax infection, namely hemodynamic instability and shock [21,23]. Interestingly, the incidence of cardiovascular instability has led some investigators to further examine the pathological and physiological effects of the toxins on the heart [22]. Despite the novelty of these studies, nearly all of them were limited to the use of the rodent model which has limitations when used for comparison to higher organisms, namely humans. The rabbit model, on the other hand, has gained recognition as a reliable animal model for studying inhalational anthrax infection, primarily due to the similar anthrax-related pathology/pathophysiology between rabbits and humans [24].

In this study we assess the hemodynamic effects, including heart rate (HR) and mean arterial pressure (MAP), of LeTx and EdTx in the rabbit model using physiologically relevant amounts of the toxins. Furthermore, we examine the degree of cardiac tissue pathology caused by LeTx which is the exotoxin historically known to bring about significant tissue destruction. We accomplish this by using telemetry (while the animals were fully conscious and unrestrained), echocardiography, histopathology, and blood analysis. To our knowledge, this is the first report describing the hemodynamic and cardiac effects of recombinant anthrax toxins in the rabbit model.

## 2. Materials and Methods

### 2.1. Animals

Specific-pathogen-free female Dutch-belted dwarf rabbits (Myrtle’s Rabbitry, Thompson Station, TN) were studied at approximately 14 weeks of age, weighing 1.5 to 2.0 kg. Upon delivery, the animals were singly housed at the University of Texas Medical Branch Animal Resources Center or the Galveston National Laboratory in stainless steel, ventilated rabbit racks (Allentown, Allentown, NJ) and allowed to acclimate for a minimum of one week before the commencement of any experimental procedures. Rabbits were fed approximately 170 g of 5321 rabbit diet (PMI LabDiet, Richmond, IN) daily and given water *ad libitum. *The animal room was maintained on a 12:12 h light:dark cycle, with the temperature range at 19 to 22 °C and the humidity between 30% and 70%. Animal procedures were conducted under protocols approved by the University of Texas Medical Branch Institutional Animal Care and Use Committee.

### 2.2. Telemetry Instrumentation and Data Acquisition

The telemetry equipment was purchased from Data Sciences International in St. Paul, MN. The implantable transmitter (model TL11M3-D70-PCTP) had two pressure leads for monitoring mean arterial pressure (MAP) and two biopotential leads for recording electrocardiogram (ECG). The system also included the Data Exchange Matrix and the RMC-1 wireless receiver. The software used for data analysis was Dataquest A.R.T. gold 4.1.

Aseptic surgical implantation of the transmitters (model TL11M3-D70-PCTP) (Data Sciences International, St. Paul, MN) was accomplished as previously described [24]. Briefly, the animals were anesthetized with ketamine (10–15 mg/kg IM) (Fort Dodge Animal Health, Fort Dodge, IA) and diazepam (0.3–0.5 mg/kg IM) (Hospira, Inc., Lake Forest, Ill), and maintenance was achieved with the use of 2–4% isoflurane (Webster Veterinary, Sterling, MA). A midline laparotomy was made at the middle 1/3 of the abdomen, while a second incision was made on the medial aspect of the left thigh. The femoral artery was isolated and cannulated with one of the two pressure catheters on the transmitter (the other pressure catheter was not used). Lastly, the catheter was tied down with stay sutures, and the animal’s incision was closed in routine fashion. The electrocardiogram (ECG) leads were exteriorized from the abdomen just cranial to the laparotomy incision. A skin incision was made on the cranial aspect of the right hemi-thorax, and a subcutaneous tunnel was made just cranial to the midline incision to the above mentioned incision site for placement of the negative lead. The positive lead was placed just cranial to the last rib. Both ECG leads was sutured into the muscle layer. The animals were allowed to recover 1 to 2 weeks before subsequent procedures.

Heart rate (HR), mean arterial pressure (MAP), and ECG were recorded continuously (every 60 s) for 24 h before challenge and for either 48 or 72 h thereafter. The recorded data for HR and MAP were used to compute a two-hour moving average. These data were in turn exported to an Excel spreadsheet for graph preparation. ECG was recorded continuously as 1 min segments.

### 2.3. Challenge Procedure

LeTx-challenged animals received recombinant LF and PA (BEI Resources, Manassas, VA) at doses of either 0.67 mg/kg and 0.24 mg/kg (low-dose) or 0.98 mg/kg and 1.95 mg/kg (high-dose), respectively. EdTx-challenged animals received recombinant EF and PA at a dose of 0.13 mg/kg and 0.24 mg/kg, respectively. The control animals received only the vehicle (PBS). Administration of the toxins or PBS, which occurred following a 24 h control period, was accomplished by intravenous injection into the animals’ marginal ear veins.

### 2.4. Measurement of Cardiac Troponin I

Blood was collected from the animals’ ears (central artery) before and after LeTx-challenge. The times of collection after challenge were 24, 48, and 72 h. Immediately following collection, the whole blood was analyzed using an iSTAT-1 handheld clinical analyzer (Abbott Point of Care, Inc., Princeton, NJ).

### 2.5. Histological Analysis

The rabbits were anesthetized and the hearts were excised 72 h post-challenge. The hearts were sectioned in a coronal plane and washed in PBS. Following, the tissue sections were perfused with 10% phosphate buffered formalin. Tissue sections were routinely processed, embedded in paraffin, sectioned at 5 µm, and stained with hematoxylin and eosin (H&E) and evaluated by light microscopy. Tissue lesions were scored based on a severity scale of minimal, mild, moderate, and marked; the scale correlated with estimates of lesion distribution and extent of tissue involvement (*i.e.*, minimal = 2–10%; mild > 10–20%; moderate > 20–50%; severe > 50). Acute inflammation indicated the presence of polymorphonuclear leukocytes (heterophils).

### 2.6. Echocardiography

Echocardiography was performed using a Sonosite Titan ultrasound (Sonosite, Bothell, WA) with an 8-MHz probe. The animals were anesthetized with 2–4% isoflurane. Echocardiography was performed 24 h prior to injection of high-dose LeTx and at 1, 2, 4, 24, and 48 h post-challenge. M-mode analysis from the right parasternal short-axis view was used to determine the left ventricular internal diameter during systole (LVIDs) and diastole (LVIDd).

### 2.7. Statistical Analysis

Data were evaluated for statistical significance using analysis of variance and linear regression procedures. Power was checked at each test. For the cTnI study, linear regression analyses were conducted to assess the significance of the observed time-related change. Both parametric and non-parametric analyses were performed, and the inferences were the same. For the echocardiography study, overall group means were compared using the inter-animal variation as the error variance. However, the pooled animal by time interaction mean square was used as the error variance to assess the significance of overall time-related means differences and for comparing group means at individual time periods. For the telemetry data, the timewise comparisons among the groups were made using Student *t*-tests with *p* < 0.05 as the significance level. Statistical calculations were made using NCSS (Kaysville, Utah).

## 3. Results

### 3.1. Changes in Heart Rate and Mean Arterial Pressure

**Figure 1:**
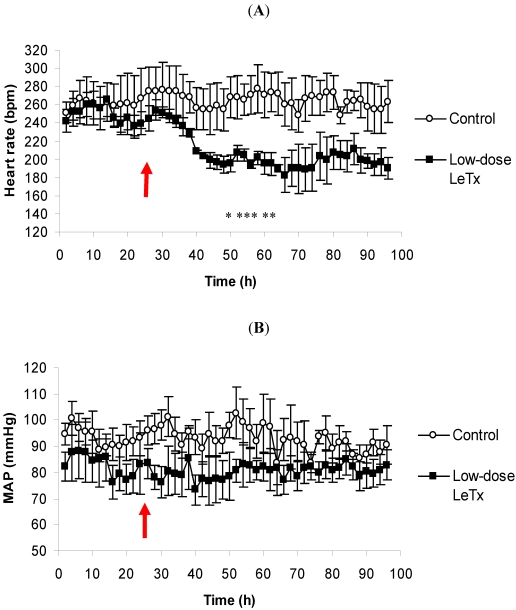
The effects of low-dose LeTx on (A) HR and (B) MAP. Rabbits (*n* = 3 per group) were intravenously injected with 0.67 mg/kg LF and 0.24 mg/kg PA following a 24 h control period. The parameters were recorded continuously, and the data are presented as a two-hour moving average. The red arrow denotes the time of challenge, and the vertical bars represent two standard errors of the means. * Indicates different from the corresponding control time-point (*p* < 0.05).

The average HR of the animals given low-dose LeTx began to fall several hours after toxin administration (Figure 1A). By 50 h, which was 26 h post-challenge, the average HR of the challenged animals was significantly lower than the control group, and it remained notably lower for the remainder of the recording period. With regards to the ECG, there were no apparent abnormalities (data not shown). Due to the association between heart rate and blood pressure, we also recorded the corresponding MAP of which there were no changes in either the control group or the low-dose LeTx-challenged group (Figure 1B).

**Figure 2:**
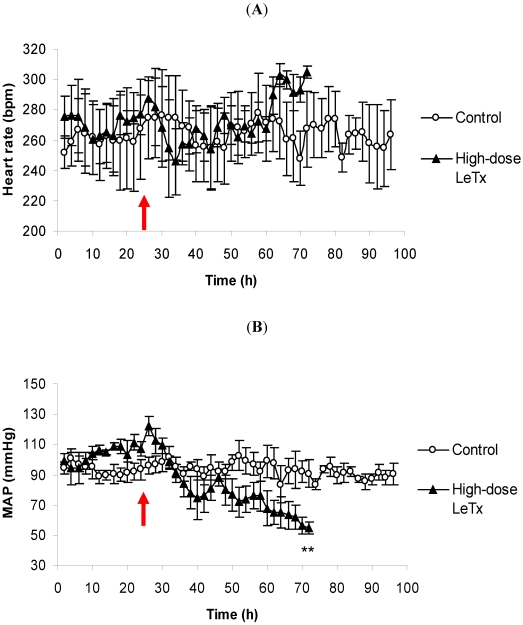
The effects of high-dose LeTx on (**A**) HR and (**B**) MAP. Rabbits (*n* = 3 per group) were intravenously injected with 0.98 mg/kg LF and 1.95 mg/kg PA following a 24 h control period. The parameters were recorded continuously, and the data are presented as a two-hour moving average. The red arrow denotes the time of challenge, and the vertical bars represent two standard errors of the means. * Indicates different from the corresponding control time-point (*p* < 0.05).

Conversely, administration of high-dose LeTx did not bring about any significant difference in the average HR of the challenged animals relative to the control group (Figure 2A). It does, however, appear that the average HR begins to decline hours after challenge, but it rebounds at approximately 35 h and reaches its peak by 72 h after which the recording was stopped and the animals were euthanized. Also, there were no apparent changes in the ECG (data not shown). The corresponding average MAP of the animals given high-dose LeTx fell dramatically following challenge and was significantly lower than the control animals at the end of the recording period (Figure 2B). The recording period for the high-dose animals was shorter than that of the low-dose animals due to the fact that by 48 h post-challenge, a couple of the high-dose animals appeared moribund and were therefore humanely euthanized.

**Figure 3:**
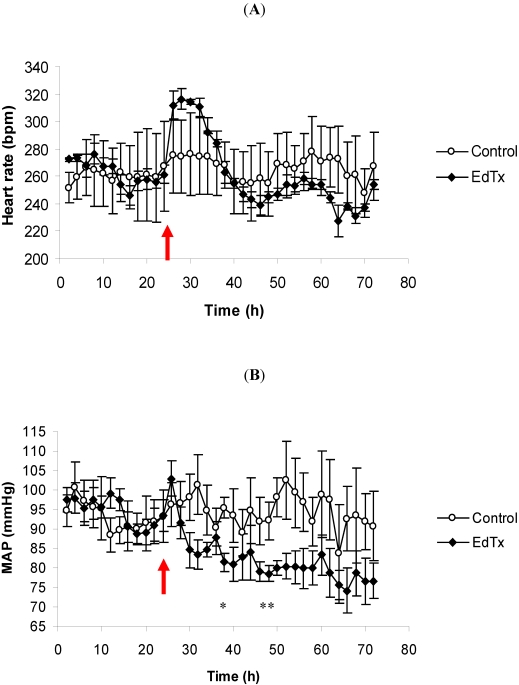
The effects of EdTx on (A) HR and (B) MAP. Rabbits (*n* = 3 per group) were intravenously injected with 0.13 mg/kg EF and 0.24 mg/kg PA following a 24 h control period. The parameters were recorded continuously, and the data are presented as a two-hour moving average. The red arrow denotes the time of challenge, and the vertical bars represent two standard errors of the means. * Indicates different from the corresponding control time-point (*p* < 0.05).

EdTx induced a tachycardic response immediately after challenge with the average HR reaching nearly 320 bpm (Figure 3A). Even though this peak HR was not statistically different from the control group, it was, however, far above the HR of the challenged animals during their 24 h control period. By approximately 16 h post-challenge (40 h), the average HR of the EdTx-challenged animals returned to or below control levels. The ECG recordings revealed no changes (data not shown). There was also a decline in the corresponding average MAP that did not rebound during the recording period (Figure 3B). By 14 h post-challenge, the MAP of the challenged animals was significantly lower than that of the control animals.

### 3.2. Cardiac Pathology

**Figure 4:**
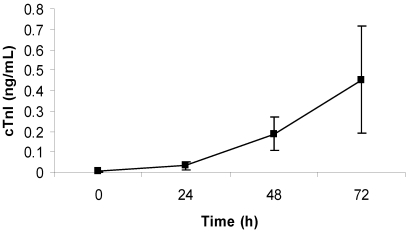
Average cTnI level in blood of rabbits challenged with low-dose LeTx. Rabbits (*n* = 5) were intravenously injected with 0.67 mg/kg LF and 0.24 mg/kg PA, and the concentration of cTnI in the blood was measured each day for 3 days after challenge (0 h was before challenge). The vertical bars represent two standard errors of the means.

**Figure 5:**
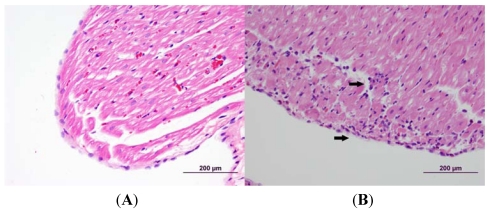
Cardiac histopathology of rabbit challenged with low-dose LeTx *versus* control rabbits. Challenged rabbits were intravenously administered 0.67 mg/kg LF and 0.24 mg/kg PA. The hearts were collected 72 h post-challenge and fixed in 10% buffered formalin. The tissues were then processed, embedded in paraffin, sectioned and analyzed by light microscopy. The arrows indicate areas of necrosis. (**A**) Control; (**B**) Low-Dose LeTx.

Animals that were challenged with low-dose LeTx showed a steady, significant (*p* < 0.05) increase in the amount of cTnI detected in the blood over time, although there was a large variation at the latest time-point (Figure 4). By 48 h post-challenge, the cTnI level rose nearly 50% (0.19 ± 0.08 ng/mL), and by 72 h post-challenge, the level rose over 100% (0.45 ± 0.26 ng/mL). Histopathological analysis revealed the presence of mild myodegeneration/necrosis and subacute inflammation with histiocytes, mononuclear cells, and heterophils present (Figure 5).

**Figure 6:**
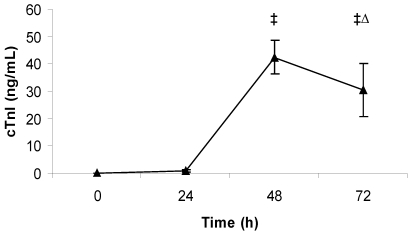
Average cTnI level in blood of rabbits challenged with high-dose LeTx. Rabbits (*n* = 5) were intravenously injected with 0.98 mg/kg LF and 1.95 mg/kg PA, and the concentration of cTnI in the blood was measured each day for 3 days after challenge (0 h was before challenge). ‡ Indicates that one or more values were above the maximum test range (50 ng/mL). ∆ indicates that *n* = 3 at this time-point. The vertical bars represent two standard errors of the means.

**Figure 7:**
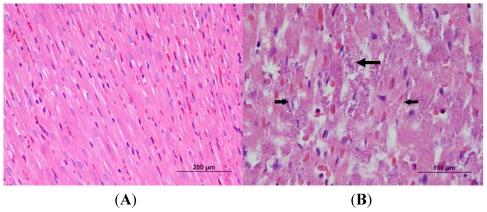
Cardiac histopathology of rabbit challenged with high-dose LeTx *versus* control rabbits. Challenged rabbits were intravenously administered 0.98 mg/kg LF and 1.95 mg/kg PA. The hearts were collected 72 h post-challenge and fixed in 10% formalin. The tissues were then processed, embedded in paraffin, sectioned and analyzed by light microscopy. The arrows indicate areas of necrosis. (**A**) Control; (**B**) High-Dose LeTx

In the case of high-dose challenged rabbits, there was a dramatic increase in the levels of cTnI, with the largest increase occurring by 48 h post-challenge (Figure 6). Important to note is that some animals had cTnI values greater than the measurable range (50 ng/mL) at 48 and 72 h post-challenge, and that blood was not collected from all the animals at 72 h post-challenge because two of the five animals had to be euthanized before the collection time. There was acute, multifocal cardiac muscle/myocyte necrosis with and without mineralization in the ventricles and the interventricular septum which was moderate in severity (Figure 7). In the second histopathology panel, the arrows indicate areas of necrosis with prominent mineralization. Moreover, acute inflammation (heterophils) in the heart was consistent in all the animals challenged with high-dose LeTx.

### 3.3. Echocardiography

**Figure 8:**
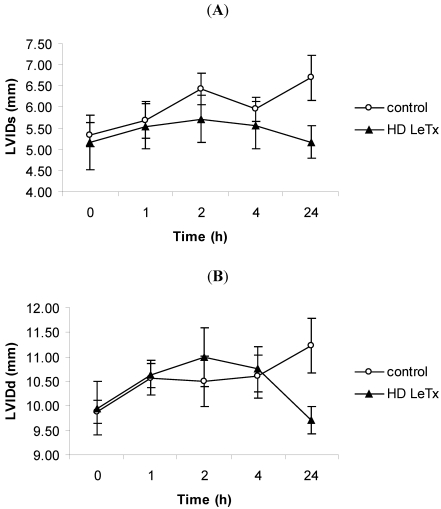
Echocardiography in rabbits challenged with high-dose LeTx. Rabbits were intravenously administered 0.98 mg/kg LF and 1.95 mg/kg PA. The left ventricular internal diameter during systole (LVIDs) (**A**) and diastole (LVIDd) (**B**) was measured at 1, 2, 4, and 24 h post-challenge. The vertical bars represent two standard errors of the means.

The internal diameter of the left ventricular cavity was measured by M-mode echocardiography in order to ascertain the presence of ventricular dilation following challenge with high-dose LeTx. Both the systolic and diastolic diameters for the high-dose LeTx-challenged animals did not change significantly (Figure 8A,B). The same analysis was performed on a separate group of animals at 48 h post-challenge which corresponds to the time at which the level of cTnI was highest, however, there was no increase in the internal diameter (data not shown).

## 4. Discussion

The amounts of toxin we chose to administer corresponds to the quantities of LeTx and EdTx measured in the sera of rabbits that were either soon to expire or had recently expired from infection initiated by challenge with virulent anthrax spores [25,26]. Our low-dose LeTx and EdTx were derived from Molin *et al*. [26], and our high-dose LeTx was derived from Mabry *et al*. [25] (our PA concentration in the high dose was lower than what was reported). In using these physiologically relevant amounts of toxin, we hoped to bring about the physiological effects that one would expect to see in the late stages of anthrax infection in the rabbit model. Also important to note is that our low-dose LeTx and EdTx were non-lethal doses, while the high-dose LeTx caused approximately 40% of the animals to become moribund (moribund animals were euthanized).

The low-dose LeTx was able to depress HR in the rabbit model without altering MAP, however, the decline did not progress past a certain level, possibly due to our use of a single injection. Interestingly, this waning HR was also reported in rats [16,22] that were administered LeTx either by a single injection or by continuous infusion. Administration of high-dose LeTx, on the other hand, caused no sustainable decrease in the average HR, but it did induce severe hypotension which progressed continually following challenge. This depression in MAP could be due to the ability of LeTx to destroy endothelial cells, thereby producing a compromised vasculature [12], as well as to its capacity to cleave intact MAPKKs that contribute to normal smooth muscle contraction [27,28]. With regards to HR, we suspect that there was no bradycardic response after high-dose LeTx-challenge because of the feedback mechanism known as reflex tachycardia whereby the heart will beat faster in order to raise blood pressure. In examining the data closely, it appears that the average HR begins to fall after high-dose LeTx-challenge, but upon decline of the average MAP, the HR rebounds. Furthermore, the average HR steadily increases until the end of the recording period which corresponds with the progressively declining MAP. This feedback mechanism may have also occurred in canines that were infused with high-dose LeTx [29]. Similar to our rabbit model, the canines showed an elevated HR concomitant with a depressed MAP following LeTx-challenge. Overall, our findings in the rabbit model, along with those reported previously in rodents [16,22], support the idea that LeTx directly alters cardiac physiology by acting as a negative chronotropic agent, and because so, could directly influence the sinoatrial (SA) node which acts as the pacemaker for the heart. The absence of any arrhythmias, which would be apparent by ECG, further suggests that the physiological effect is restricted primarily to the SA node and not at the myocardial tissue. Further evidence for the direct effect of LeTx on the heart was recently reported in an isolated perfused rat heart model [30]. Here, the investigators demonstrated that a high dose of LeTx can depress HR in an isolated heart as well. Nonetheless, with regards to an *in vivo* model, the toxin could also depress HR by inducing changes in the nervous system (*i.e.*, parasympathetic stimulation). Unfortunately, the precise role of MAPKKs, the target of LeTx, in cardiac contraction is not completely known.

The hemodynamic effects of EdTx that we present here in the rabbit model parallel those reported in both the rodent model [22] and canine model [29]. Moreover, in the isolated rat heart model, there is also a transient increase in HR [30]. The changes are due to the toxin’s ability to produce large amounts of intracellular cAMP. This second messenger increases heart rate by indirectly stimulating Ca^2+^ release from the sarcoplasmic reticulum [31], and it lowers blood pressure by relaxing smooth muscles that line the vasculature [32].

Due to the cytotoxicity of LeTx in various organs of rodents [17,19], we sought to determine the pathological effects of the toxin on cardiac tissue which would in turn alter hemodynamics. The pathology we observed in the cardiac tissue of the low-dose LeTx-challenged animals was mild, yet notable, and marked by cardiomyocyte necrosis and inflammation. The same was seen in the high-dose LeTx-challenged rabbits but to a greater extent. The incidence of mineralization, which is evidence of the necrotic process, and the cTnI levels distinguished the effects of the low-dose and high-dose LeTx. Unfortunately, much of the pathology observed, especially with the high-dose LeTx-challenged animals, could be due to tissue hypoperfusion which would result from the severe hypotension, but the fact that even the low-dose LeTx-challenged animals showed a significant increase in cTnI over time with normal MAP discounts this. Instead, it suggests that the toxin can damage cardiac tissue much like other organ tissues. After all, past reports show that MAPK ERK 1/2 inhibition promotes stress-induced apoptosis and heart failure [33].

Despite what has been reported previously in rodents after LeTx challenge [19,22], we did not observe left ventricular systolic/diastolic dilation in the rabbits following challenge with high-dose LeTx. For the animals that we used in this analysis, we administered only the higher dose of LeTx due to the possibility that any change in myocardial structure, as in the case of ventricular dilation, might not occur as readily as a functional change (such as with HR), and therefore would require more toxin. However, there still was no significant change with the higher dose. This discrepancy in ventricular dilation between our rabbit model and the rodent model can possibly be attributed to the amount of toxin administered. Rabbits might require much more LeTx (to achieve a higher mortality) to show the cardiac dilation similar to that of rodents. Then again, in looking at the doses of LeTx on a per-kg basis, our rabbits received more toxin than the rodents involved in previous studies, with our high dose being much more. If we were to challenge our rabbits with similar doses (on a per kg basis) as the rodents in past reports, we possibly would have had 0% morbidity. This implies that, when comparing different animal models, body size alone is not sufficient for determining the quantity of toxin that would propagate notable pathophysiology.

## 5. Conclusions

In this report, we present certain hemodynamic effects of anthrax toxins in the rabbit model. Similar studies have been performed in the past, however, they primarily employed the rodent model which at times has inadequacies as a model system, especially when used to make generalizations about higher organisms. We find that LeTx and EdTx can in fact alter hemodynamics in rabbits similar to that of rodents, however, our results suggest that in the case of LeTx, there is a difference with regards to the level of susceptibility between the two models as based on the amount of LeTx administered on a per kg basis. Another difference that we observed was the absence of ventricular dilation in our rabbit model. We hope these findings add to what is known about anthrax pathophysiology in animal models.
